# Observational and genetic association of non-alcoholic fatty liver disease and calcific aortic valve disease

**DOI:** 10.3389/fendo.2024.1421642

**Published:** 2024-07-09

**Authors:** Qing-Yun Hao, Yu-Hong Zeng, Ying Lin, Jing-Bin Guo, Shi-Chao Li, Ping-Zhen Yang, Jing-Wei Gao, Ze-Hua Li

**Affiliations:** ^1^ Department of Cardiology, Laboratory of Heart Center, Zhujiang Hospital, Southern Medical University, Guangzhou, China; ^2^ Medical Apparatus and Equipment Deployment, Zhujiang Hospital, Southern Medical University, Guangzhou, China; ^3^ Department of Endocrinology, Sun Yat-sen Memorial Hospital, Sun Yat-sen University, Guangzhou, China; ^4^ Department of Organ Transplantation, Zhujiang Hospital, Southern Medical University, Guangzhou, China; ^5^ Department of Cardiology, Sun Yat-sen Memorial Hospital, Sun Yat-sen University, Guangzhou, China

**Keywords:** calcific aortic valve disease, non-alcoholic fatty liver disease, aortic valve calcification, Mendelian randomization analyses, risk

## Abstract

**Background:**

Non-alcoholic fatty liver disease (NAFLD) has emerged as a predominant driver of chronic liver disease globally and is associated with increased cardiovascular disease morbidity and mortality. However, the association between NAFLD and calcific aortic valve disease remains unclear. We aimed to prospectively investigate the association between NAFLD and incident aortic valve calcification (AVC), as well as its genetic relationship with incident calcific aortic valve stenosis (CAVS).

**Methods:**

A *post hoc* analysis was conducted on 4226 participants from the Multi-Ethnic Study of Atherosclerosis (MESA) database. We employed the adjusted Cox models to assess the observational association between NAFLD and incident AVC. Additionally, we conducted two-sample Mendelian randomization (MR) analyses to investigate the genetic association between genetically predicted NAFLD and calcific aortic valve stenosis (CAVS), a severe form of CAVD. We repeated the MR analyses by excluding NAFLD susceptibility genes linked to impaired very low-density lipoprotein (VLDL) secretion.

**Results:**

After adjustment for potential risk factors, participants with NAFLD had a hazard ratio of 1.58 (95% CI: 1.03–2.43) for incident AVC compared to those without NAFLD. After excluding genes associated with impaired VLDL secretion, the MR analyses consistently showed the significant associations between genetically predicted NAFLD and CAVS for 3 traits: chronic elevation of alanine aminotransferase (odds ratio = 1.13 [95% CI: 1.01–1.25]), imaging-based NAFLD (odds ratio = 2.81 [95% CI: 1.66–4.76]), and biopsy-confirmed NAFLD (odds ratio = 1.12 [95% CI: 1.01–1.24]). However, the association became non-significant when considering all NAFLD susceptibility genes.

**Conclusions:**

NAFLD was independently associated with an elevated risk of incident AVC. Genetically predicted NAFLD was also associated with CAVS after excluding genetic variants related to impaired VLDL secretion.

## 1 Introduction

Non-alcoholic fatty liver disease (NAFLD) is prevalent in approximately 32% of adults and afflicts half of those with type 2 diabetes mellitus (1, 2), NAFLD has become the most common cause of chronic liver disease worldwide. NAFLD is a clinical-pathological syndrome, ranges from simple steatosis to non-alcoholic steatohepatitis, with potential progression to advanced stages such as fibrosis, cirrhosis, liver failure, and hepatocellular carcinoma (3). Patients with NAFLD have a substantially higher mortality rate than the non-NAFLD controls, with cardiovascular disease (CVD) being the primary reason for death (4).

Calcific aortic valve disease (CAVD) comprises aortic valve sclerosis and calcific aortic valve stenosis (CAVS), characterized by calcium build-up on the aortic valve (5). Ranking as the third leading cause of CVD globally, CAVD affects about 25% of those over 65 years (6). The Global Burden of Disease Study in 2017 showed that the disability-adjusted life years related to CAVD increased by 101% from 1990 to 2017 (7). CAVS dominates as the chief valvular disease among Western populations and is responsible for the highest valve-related mortality in the United States (8). With a prevalence exceeding 2% in those over 65, projections suggested a twofold increase in aortic valve replacement by 2050 (9). Aortic valve calcification (AVC) is now perceived as an actively regulated biological process that shares many similar risk factors with coronary artery atherosclerosis (10). Additionally, it has been demonstrated that NAFLD is significantly associated with a higher risk of atherosclerosis (11). Previous studies have established a link between NAFLD and coronary artery calcification (12); and showed an cross-sectional association between NAFLD and aortic and mitral valve calcification (13, 14). While the prospective and causal relationship between NAFLD and CAVD remains unknown.

Mendelian randomization (MR) analysis has become prevalent in studying the causal relationship between various exposure factors and outcomes of interest. As genetic variants that increase susceptibility or protect against the exposure of interest are randomly assigned at conception, they can be used as instrumental variables (IVs) in MR analysis. Thus, MR analysis is often regarded as similar to a randomized controlled trail but with lower susceptibility to reverse causation and confounding factors (15). However, there has been no MR analysis on the association between NAFLD and CAVD yet.

This study aimed to investigate both the observational and genetic association between NAFLD and CAVD. To achieve this objective, we initially examined the observational association between NAFLD and incident AVC using data from the Multi-Ethnic Study of Atherosclerosis (MESA). Then, we conducted two-sample MR analyses utilizing recently obtained genome-wide association study (GWAS) data to explore the association between genetically predicted NAFLD and CAVS.

## 2 Methods

### 2.1 Study design and population

The MESA is an ongoing, prospective, population-based observational study aimed at investigating early, or subclinical, atherosclerosis and CVD dynamics, with previously published details covering examinations and protocols (16). From July 2000 to September 2002, 6814 participants were enrolled in 6 United States field centers. Before the study initiation, all participants underwent screening to ensure they were between 45 and 84 years old and had no clinical CVD. Every participant provided informed consent, and the Institutional Review Boards approved the study protocols of all participating institutions.

**Figure 1:**
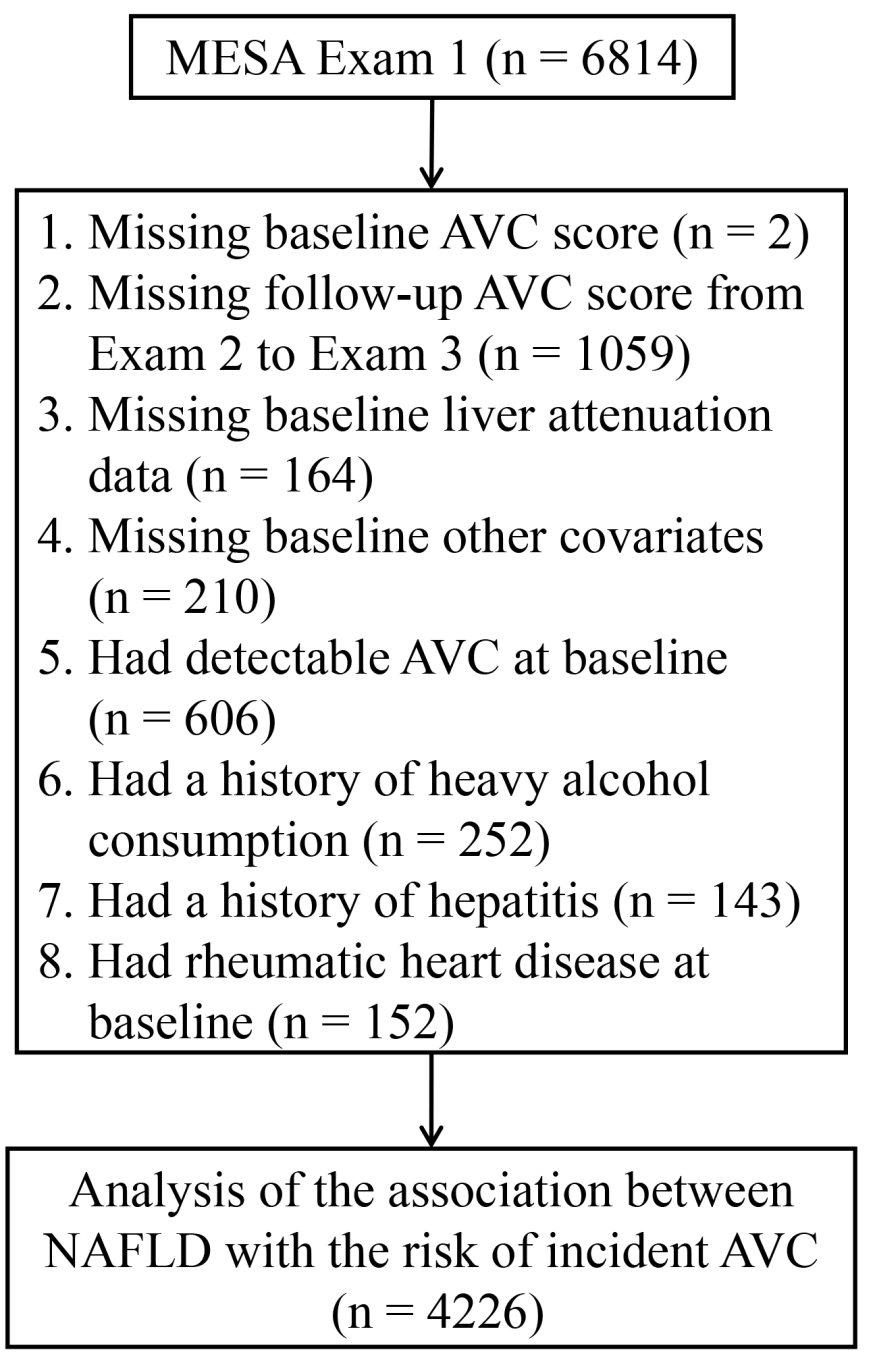
Flowchart for selecting the participants from the MESA for analysis. AVC, aortic valve calcification; MESA, Multi-Ethnic Study of Atherosclerosis; NAFLD, non-alcoholic fatty liver disease.

We included participants who had baseline (Exam 1: 2000–2002) and follow-up (Exam 2: 2002–2004 and Exam 3: 2004–2005) AVC measurements in the present study. We excluded those with missing data on AVC score at baseline (n = 2), or during follow-up AVC score (n = 1059), baseline liver attenuation data (n = 164), covariates data (n = 210), or those had detectable AVC at baseline (n = 606), had a history of heavy alcohol consumption (defined as > 7 drinks/week for women and > 14 drinks/week for men, n = 252) (17), had a history of hepatitis (n = 143), or had a history of rheumatic heart disease at baseline (n = 152). Consequently, the final study sample consisted of 4226 participants (**Figure 1**).

### 2.2 Measurements of liver fat

The details of liver fat measurement in the MESA study have been described previously (18). During the initial examination, every participant underwent 2 successive non-enhanced cardiac-gated computed tomography (CT) scans, which provided sufficient liver imaging to determine fatty liver. Prior research has shown a negative correlation between liver attenuation on CT scans and liver fat deposition on liver biopsy, indicating that CT scanning is a valuable non-invasive approach to detecting fatty liver (19). Briefly, baseline cardiac CT scans were utilized to measure hepatic attenuation values (Hounsfield Units, HU) using a region of interest of ≥ 100 mm^2^ in area. NAFLD was defined as liver attenuation value < 51 HU after ruling out alternative factors contributing liver fat accumulation in individuals without heavy alcohol use. This threshold is equivalent to a liver-to-spleen ratio of < 1.0, which indicates at least mild NAFLD (17, 20).

### 2.3 Measurements of AVC

Calcified lesions on aortic valve leaflets and the aortic root were characterized as AVC in the MESA study, assessed by either electron-beam CT or multi-detector row helical CT depending on the study site to detect cardiovascular calcification (21). All scans underwent centralized analysis at the Harbour-UCLA Research and Education Institute, Los Angeles, CA, ensuring low intrareader and interscan variability (4.4% and 9.7%, respectively) in AVC scoring (21, 22). AVC score is calculated using the Agatston method, where the calcification score of each lesion is determined by multiplying the lesion area by a density factor derived from the maximum HU within this area, the total AVC score is determined by summing the Agatston score of all individual lesions (23). AVC score > 0 indicates the presence of AVC, while AVC score = 0 indicates the absence of AVC (22). Follow-up measurements for AVC were conducted during Exam 2 (2002–2004) or Exam 3 (2004–2005). While some participants underwent additional CT scans at Exam 4 and/or Exam 5 for assessing coronary artery calcification, these examinations were not included for the present analysis due to lack of AVC data. Incident AVC was defined as AVC score > 0 during follow-up in individuals who had no AVC (AVC score = 0) at the baseline examination (24).

### 2.4 Measurements of other covariates

Participants’ baseline characteristics were obtained at Exam 1. Participants provided self-reported information on age, sex, ethnicity, smoking status, drinking status, and medication history. Physical activity was measured using the MESA typical week physical activity survey. Body mass index (BMI) was calculated as weight in kilograms divided by height in meters squared (kg/m^2^). Hypertension was identified based on systolic blood pressure readings of 140 mmHg or higher, diastolic blood pressure readings of 90 mmHg or higher, or a record of antihypertensive drug usage in the preceding two weeks. Diabetes was defined by a fasting glucose level of 126 mg/dL or higher, or a self-reported physician’s diagnosis of diabetes, or any utilization of antidiabetic drugs. Detailed descriptions of measurements, including plasma total cholesterol, high-density lipoprotein cholesterol, triglycerides, low-density lipoprotein cholesterol, fasting glucose, estimated glomerular filtration rate, and C-reactive protein, have been previously published (25).

### 2.5 GWAS data sources for NAFLD

Gene-exposure data were derived from a GWAS conducted on the chronic elevation of alanine aminotransferase (cALT) in the Million Veteran Program, which was a large-scale study that seeks to pinpoint genetic and non-genetic risk factors for various health conditions in US veterans. Within this study, NAFLD was defined as alanine transaminase levels surpassing gender-specific thresholds (30 U/L for females and 40 U/L for males) on 2 occasions separated by at least six months within a two-year frame, excluding other liver diseases (26). The specific design, initial demographics, and quality-control procedures of this study have been detailed previously (27). The study included 128,187 controls and 90,408 NAFLD cases from 4 ancestral groups, including Asian-Americans (0.9%), Hispanic-Americans (6.9%), African-Americans (17.1%), and European-Americans (75.1%). Among these participants, 77 independent single nucleotide polymorphisms (SNPs) were identified as having genome-wide significance (*P* < 5×10^-8^) (28). Further, validation was performed on 22 SNPs in 1 external cohort and 36 SNPs in the other one. The first cohort consisted of 44,289 individuals with liver fat measured through CT or magnetic resonance imaging, while the second cohort consisted of 64,182 individuals with biopsy-confirmed NAFLD, respectively (28).

### 2.6 GWAS data sources for CAVS

The CAVS-related GWAS data were provided by the FinnGen study (https://r9.finngen.fi/), a large public-private partnership project launched in Finland in 2017 to gather and examine health and genome information from 500,000 Finish biobank participants. The GWAS of CAVS in FinnGen included 9153 cases and 368,124 controls and analyzed approximately 20,170,236 SNPs. The FinnGen study defined CAVS using the ICD-9 codes 4241B and 4241C and ICD-10 codes I35.0 and I35.2.

### 2.7 Selection of genetic instruments

To determine the genetic association between NAFLD and CAVS, we employed 3 IVs sets, including (1) all 77 SNPs associated with cALT; (2) 22 cALT-associated SNPs that showed directional concordance and nominal significance in the imaging cohort, with Z-scores from the imaging data used for analyses; (3) 36 cALT-associated SNPs that showed directional concordance and nominal significance in the biopsy cohort, with effect estimates from the biopsy data used for analyses (**Supplementary Table 1**) (28). To ensure the precision of the outcomes, we reanalyzed all sets by removing the NAFLD susceptibility genes linked to a decline in very low-density lipoprotein (VLDL) secretion (APOE, BCL7B, MTTP, TM6SF2, and PNPLA3), based on the information from PhenoScanner and Pubmed search.

The selection of valid IVs in this study involved strict criteria. SNPs that exhibited linkage disequilibrium with an r^2^ < 0.001, and < 1 MB from the index variant, or displayed palindromic patterns with intermediate allele frequencies were removed from the analyses. Additionally, SNPs that were not present in the outcome GWAS dataset were excluded. The strength of IVs was evaluated by calculating F statistic (29), and only IVs with an F statistic > 10 were deemed valid and reliable for NAFLD analyses.

### 2.8 Statistical analyses

Continuous variables conforming to a normal distribution were presented as mean ± SD, while non-normally distributed continuous variables were given as median (interquartile range). Categorical variables were presented as numbers (percentages). Student’s t-test or Mann-Whitney U-test was used for continuous variables, depending on the distribution. The Chi-square test was applied for categorical variables. Adjusted Cox models were employed to examine the association between baseline NAFLD status and the risk of incident AVC, providing hazard ratios (HR) and 95% CIs. The statistical analyses were conducted using SPSS version 26. Statistical significance was determined using a two-sided *P* value of < 0.05.

The methodology for MR analysis was anchored in 3 fundamental assumptions (30). The primary analyses of our two-sample MR analyses employed inverse variance weighted (IVW) method with a random-effect model. To evaluate heterogeneity, we calculated Cochran’s Q statistic and conducted leave-one-out analyses. Horizontal pleiotropy was gauged via MR-Egger intercept test. If the *P* value from MR-Egger intercept test > 0.05, it was considered as no evidence of horizontal pleiotropy, and the estimation from IVW method was deemed the most reliable (31). We additionally performed further analyses using more rigorous criteria: (1) simple median method, even if half of the genetic instruments are invalid, the effect estimate can still be generated (28); (2) penalised weighted median method, which decreases the influence of genetic variants with heterogeneity, effectively mitigating the influence of outliers (28); (3) MR-Egger regression method, which permits pleiotropic effects for all genetic variants, with the condition that these effects are independent of the association between the variant and exposure (32); (4) weighted mode, which generates consistent results even if most IVs were invalid (33). For added precision, MR-PRESSO analyses were applied to address heterogeneity, excluding SNPs that significantly added to observed heterogeneity (NbDistribution = 10,000). All two-sample MR analyses were conducted using the TwoSampleMR package in the R 4.0.1.

## 3 Results

### 3.1 Baseline characteristics between participants with and without NAFLD

The analyses were conducted on 4226 participants from the MESA study (**Figure 1**). At baseline, the average age was 60.8 ± 10.0 years, 1879 (44.5%) were men, 1545 (36.6%) were Caucasian, 520 (12.3%) were Chinese, 1211 (28.7%) were African American, 950 (22.5%) were Hispanic, 1729 (40.9%) had hypertension, 474 (11.2%) had diabetes mellitus, and 645 (15.3%) had NAFLD (**Table 1**). The participants were then categorized into two groups depending on whether they had NAFLD at baseline. Individuals with NAFLD were younger (59.0 *vs.* 61.1 years), more Hispanic ethnicity (38.1% *vs.* 19.7%), and more non-drinkers (26.5% *vs.* 20.6%). Moreover, they exhibited increased BMI (32.0 *vs.* 27.8 kg/m^2^), systolic blood pressure (127.6 *vs.* 124.5 mmHg), diastolic blood pressure (72.9 *vs.* 71.6 mmHg), triglycerides (163.8 *vs.* 117.6 mg/dL), fasting glucose (109.3 *vs.* 93.5 mg/dL), estimated glomerular filtration rate (82.4 *vs.* 78.4 ml/min/1.73 m^2^), and C-reactive protein (3.4 *vs.* 1.7 mg/L) levels, while their high-density lipoprotein cholesterol (45.4 *vs.* 52.3 mg/dL), low-density lipoprotein cholesterol (114.6 *vs.* 118.3 mg/dL), and physical activity (630.0 *vs.* 915.0 MET-min/wk) levels were lower (**Table 1**). Additionally, participants with NAFLD exhibited a higher prevalence of diabetes mellitus (23.7% *vs.* 9.0%) and hypertension (47.8% *vs.* 39.7%) and a greater likelihood of using antihypertensive (40.0% *vs.* 32.4%) and hypoglycemic (16.6% *vs.* 7.0%) medications than those without (**Table 1**).

**Table 1:** Baseline characteristics of participants stratified by status of NAFLD.

Clinical characteristic	Total (n = 4226)	No NAFLD (n = 3581)	NAFLD (n = 645)	*P* value
Liver attenuation, HU	60.2 ± 11.7	63.8 ± 7.6	40.1 ± 9.8	< 0.001
Age, years	60.8 ± 10.0	61.1 ± 10.1	59.0 ± 8.9	< 0.001
Male, n (%)	1879 (44.5)	1582 (44.2)	297 (46.0)	0.389
Race, n (%)				< 0.001
Caucasian	1545 (36.6)	1341 (37.4)	204 (31.6)	
Chinese	520 (12.3)	441 (12.3)	79 (12.2)	
African American	1211 (28.7)	1095 (30.6)	116 (18.0)	
Hispanic	950 (22.5)	704 (19.7)	246 (38.1)	
BMI, kg/m^2^	28.4 ± 5.5	27.8 ± 5.1	32.0 ± 6.1	< 0.001
SBP, mmHg	125.0 ± 20.6	124.5 ± 20.8	127.6 ± 18.9	< 0.001
DBP, mmHg	71.8 ± 10.1	71.6 ± 10.1	72.9 ± 10.1	0.012
Smoking status, n (%)				0.473
Never smoker	2241 (53.0)	1892 (52.8)	349 (54.1)	
Former smoker	1479 (35.0)	1251 (34.9)	228 (35.3)	
Current smoker	506 (12.0)	438 (12.2)	68 (10.5)	
Drinking status,n (%)				0.002
Never drinker	907 (21.5)	736 (20.6)	171 (26.5)	
Former drinker	916 (21.7)	793 (22.1)	123 (19.1)	
Current drinker	2403 (56.9)	2052 (57.3)	351 (54.4)	
Physical activity, MET-min/wk	840.0 (185.6–2070.0)	915.0 (210.0–2100.0)	630.0 (0–1665.0)	< 0.001
Hypertension, n (%)	1729 (40.9)	1421 (39.7)	308 (47.8)	< 0.001
Diabetes mellitus, n (%)	474 (11.2)	321 (9.0)	153 (23.7)	< 0.001
Antihypertensive medication, n (%)	1420 (33.6)	1162 (32.4)	258 (40.0)	< 0.001
Hypoglycemic medication, n (%)	357 (8.4)	250 (7.0)	107 (16.6)	< 0.001
Lipid-lowering medication, n (%)	635 (15.0)	530 (14.8)	105 (16.3)	0.338
TC, mg/dL	193.9 ± 33.8	194.1 ± 33.7	192.7 ± 34.2	0.510
TG, mg/dL	124.6 ± 64.7	117.6 ± 60.4	163.8 ± 73.3	< 0.001
HDL-C, mg/dL	51.2 ± 14.5	52.3 ± 14.7	45.4 ± 11.7	< 0.001
LDL-C, mg/dL	117.8 ± 30.6	118.3 ± 30.6	114.6 ± 30.5	0.010
Fasting glucose, mg/dL	95.9 ± 27.5	93.5 ± 24.0	109.3 ± 39.3	< 0.001
eGFR, ml/min/1.73m^2^	79.0 ± 15.7	78.4 ± 15.6	82.4 ± 15.8	< 0.001
CRP, mg/L	1.9 (0.8–4.3)	1.7 (0.8–3.9)	3.4 (1.5–6.6)	< 0.001

Data are presented as mean ± SD for normal distribution continuous variables, median (25^th^–75^th^) for non-normally distributed continuous variables, and n (%) for categorical variables. BMI, body mass index; CRP, C-reactive protein; DBP, diastolic blood pressure; eGFR, estimate glomerular filtration rate; HDL-C, high-density lipoprotein cholesterol; HU, Hounsfield units; LDL-C, low-density lipoprotein cholesterol; NAFLD, non-alcoholic fatty liver disease; SBP, systolic blood pressure; TC, total cholesterol; TG, triglycerides.

### 3.2 Risk of incident AVC for NAFLD groups

Over an average follow-up period of 2.4 ± 0.9 years, 169 (4.0%) incident AVC cases were observed (**Table 2**). In the fully-adjusted model, participants with NAFLD had a significantly higher risk of AVC incidence than those without (HR = 1.58 [95% CI: 1.03–2.43]; *P* = 0.038; **Table 2**). When participants were stratified by age (< 60 or ≥ 60 years), sex (male or female), ethnicity (Caucasian, Chinese, African American, or Hispanic), and BMI (< 28 or ≥ 28 kg/m^2^), the association between NAFLD status and the risk of AVC incidence remained consistent among all the subgroups (all *P* for interaction > 0.05, **Supplementary Figure 1**).

**Table 2:** Adjusted associations between NAFLD at liver attenuation values less than 51 and AVC incident.

	Events/No. at risk	Model 1 HR (95% CI)	*P* value	Model 2 HR (95% CI)	*P* value	Model 3 HR (95% CI)	*P* value
No NAFLD	138/3581	Reference	–	Reference	–	Reference	–
NAFLD	31/645	1.66 (1.11–2.49)	0.013	1.57 (1.02–2.41)	0.039	1.58 (1.03–2.43)	0.038

Model 1: adjusted for age, race and sex.

Model 2: adjusted for Model 1 covariates plus BMI, drinking status, physical activity, SBP, and smoking status.

Model 3: adjusted for Model 2 covariates plus CRP, fasting glucose, hypertension, hypoglycemic medication use, LDL-C, and lipid-lowering medication use.

AVC, aortic valve calcification; BMI, body mass index; CRP, C-reactive protein; CI, confidence interval; HR, hazard ratio; LDL-C, low-density lipoprotein cholesterol; NAFLD, non-alcoholic fatty liver disease; SBP, systolic blood pressure.

### 3.3 Genetic association between NAFLD and CAVS

The GWAS identified 77 cALT-associated SNPs, of which 6 SNPs were unavailable within CAVS dataset, and 1 SNP was associated with CAVS. An additional 18 SNPs were removed due to being either linkage disequilibrium or palindromic, resulting in 52 independent SNPs that were used as IVs (**Supplementary Tables 1**, **2**), with a mean F statistic of 556.9.

**Figure 2:**
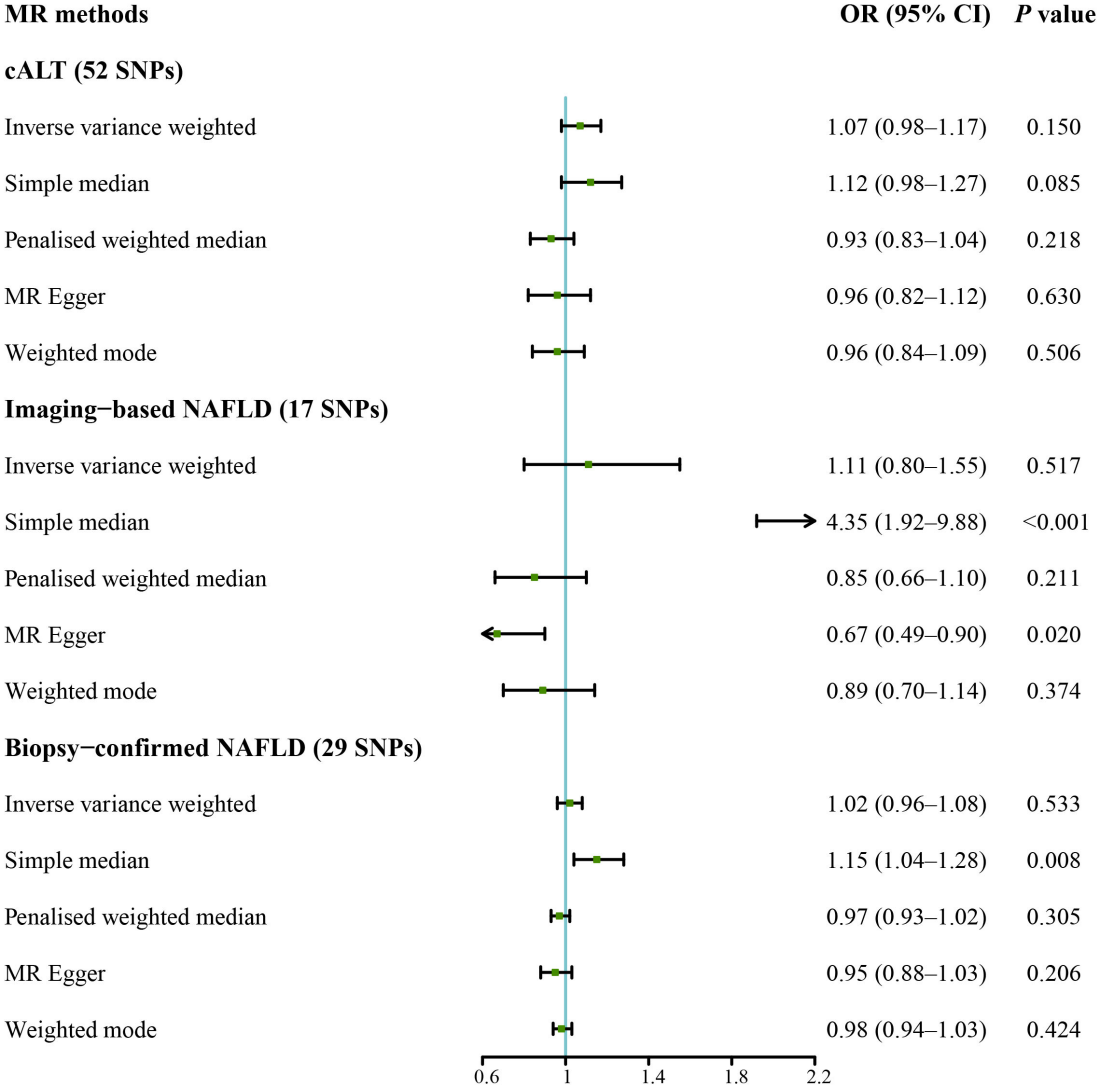
Forest plot of MR results of NAFLD with CAVS. cALT, chronic elevation of alanine transaminase; CAVS, calcific aortic valve stenosis; CI, confidence interval; MR, Mendelian randomization; NAFLD, non-alcoholic fatty liver disease; OR, odds ratio; SNPs, single nucleotide polymorphisms.

**Figure 3:**
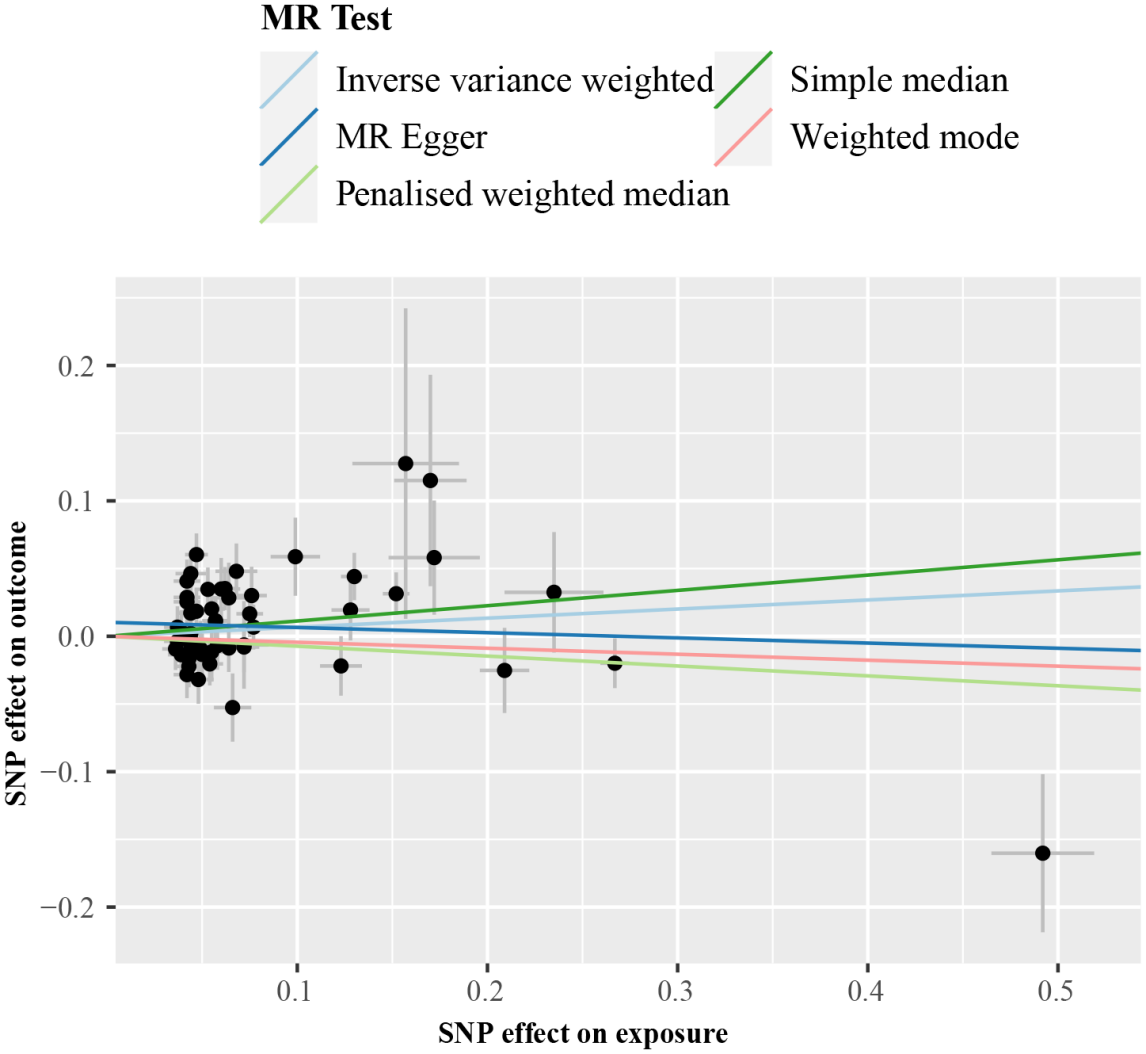
Scatter plot from genetically predicted cALT on CAVS. cALT, chronic elevation of alanine transaminase; CAVS, calcific aortic valve stenosis; MR, Mendelian randomization; SNP, single nucleotide polymorphism.

IVW analysis detected a non-significant association between genetically predicted cALT and an increased risk of CAVS (odds ratio = 1.07 [95% CI: 0.98–1.17]; *P* = 0.150; **Figure 2**). A similar trend was observed in other sensitivity analyses (**Figures 2**, **3**). Although Cochran’s Q test detected heterogeneity (Q: 101.0, *P* < 0.001), heterogeneity was considered acceptable within the context of random-effects IVW method (28). MR-Egger intercept test showed a nonsignificant intercept (*P* = 0.108), indicating the absence of pleiotropy. No outliers were identified in leave-one-out plot (**Supplementary Figure 6**). Furthermore, MR-PRESSO identified 2 outliers, although the exclusion of outliers did not substantially affect the result (odds ratio = 1.07 [95% CI: 0.97–1.17]; *P* = 0.173).

IVW analyses conducted on additional NAFLD-related traits, which included both imaging-based NAFLD (using 17 SNPs, **Supplementary Tables 1**, **3**) and biopsy-confirmed NAFLD (using 29 SNPs, **Supplementary Tables 1**, **4**), were non-significant (**Figure 2**). Consistent results were obtained using penalised weighted median and weighted mode for both traits (**Figure 2**, **Supplementary Figures 2**, **3**). MR-Egger intercept test revealed a notable intercept for both traits (*P* < 0.05), suggesting the presence of potential pleiotropy. No outliers were identified in leave-one-out plots (**Supplementary Figures 7**, **8**). MR-PRESSO identified several outliers for both traits, but there was no significant difference in the causal estimates before and after removing outliers.

### 3.4 Genetic association between NAFLD and CAVS after excluding impaired VLDL secretion-associated genes

**Figure 4:**
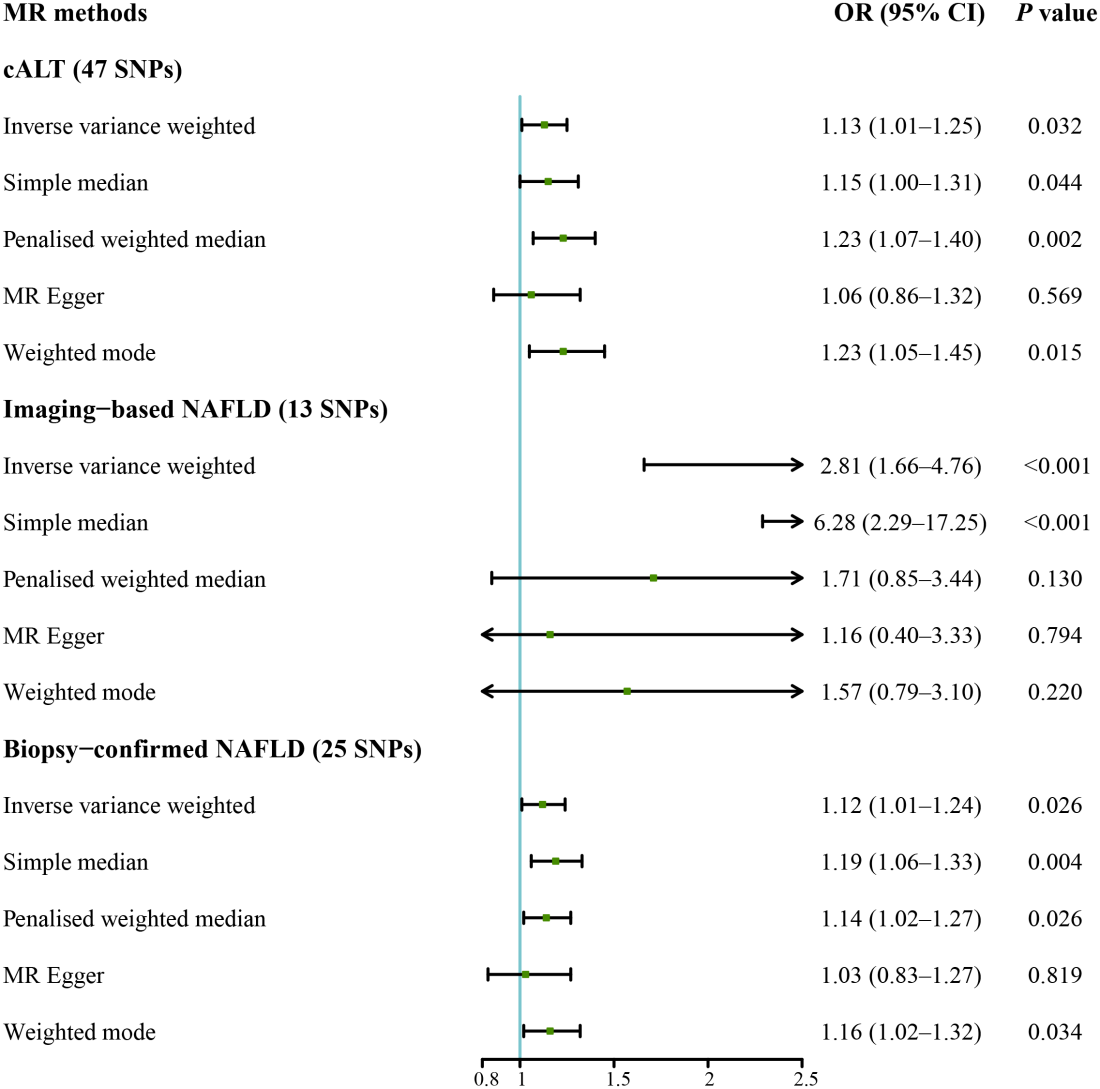
Forest plot of MR results of NAFLD with CAVS after exclusion of genes associated with impaired VLDL secretion. cALT, chronic elevation of alanine transaminase; CAVS, calcific aortic valve stenosis; CI, confidence interval; MR, Mendelian randomization; NAFLD, non-alcoholic fatty liver disease; OR, odds ratio; SNPs, single nucleotide polymorphisms; VLDL, very low-density lipoprotein.

**Figure 5:**
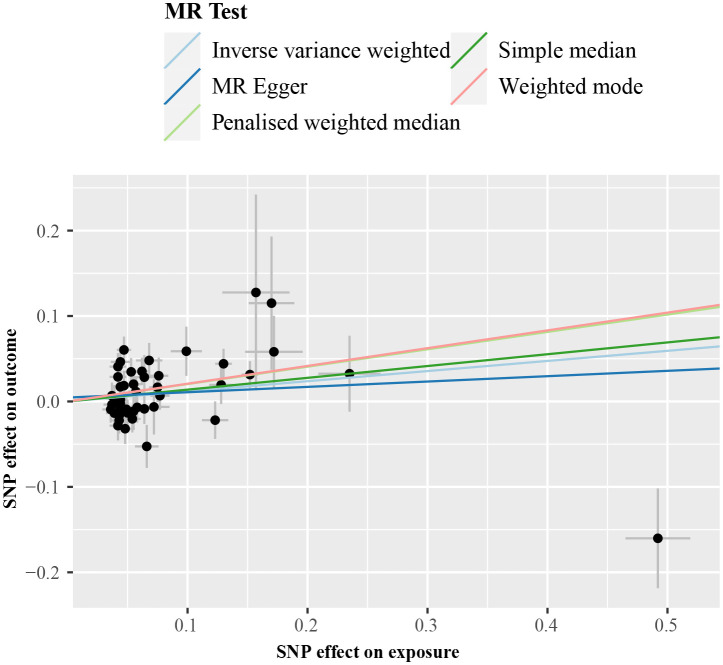
Scatter plot from genetically predicted cALT on CAVS after exclusion of genes associated with impaired VLDL secretion. cALT, chronic elevation of alanine transaminase; CAVS, calcific aortic valve stenosis; MR, Mendelian randomization; SNP, single nucleotide polymorphism; VLDL, very low-density lipoprotein.

We subsequently repeated the analyses after excluding genes that are associated with impaired VLDL secretion (APOE, BCL7B, MTTP, TM6SF2, and PNPLA3; F statistic: 465.2; **Supplementary Table 2**). IVW method with the remaining 47 SNPs showed a statistically significant association between cALT-related SNPs and the risk of CAVS (odds ratio = 1.13 [95% CI: 1.01–1.25]; *P* = 0.032; **Figure 4**). Similar associations were found when simple median, penalised weighted median, and weighted mode methods were applied, with odds ratios of 1.15 (95% CI: 1.00–1.31), 1.23 (95% CI: 1.07–1.40), and 1.23 (95% CI: 1.05–1.45), respectively (**Figures 4**, **5**). Heterogeneity was observed with Cochran’s Q test (Q: 94.4, *P* < 0.001). MR-Egger intercept test revealed a non-significant intercept (*P* = 0.554). No outliers were identified in leave-one-out plot (**Supplementary Figure 9**). However, MR-PRESSO identified 2 outliers, and the exclusion these SNPs did not substantially affect the result (odds ratio = 1.17 [95% CI: 1.06–1.29]; *P* = 0.003).

IVW MR analysis for the imaging data, including the remaining 13 SNPs after removing genes linked to a decline in VLDL secretion (F statistic: 25.9; **Supplementary Table 3**) showed statistically significant association between genetically predicted imaging-based NAFLD and risk of CAVS (odds ratio = 2.81 [95% CI: 1.66–4.76]; *P* < 0.001; **Figure 4**). Similar result was observed with the simple median method (odds ratio = 6.28 [95% CI: 2.29–17.25]; *P* < 0.001; **Figure 4**, **Supplementary Figure 4**). No heterogeneity was detected by Cochran’s Q test (Q: 11.5, *P* = 0.399), and MR-Egger intercept test demonstrated a nonsignificant intercept (*P* = 0.091). No outliers were identified in leave-one-out plot (**Supplementary Figure 10**), and MR-PRESSO method did not detect any outliers.

IVW analysis for the biopsy-confirmed NAFLD, including 25 SNPs (F statistics: 309.1; **Supplementary Table 4**), showed a statistically significant association between biopsy-confirmed NAFLD and increased risk of CAVS (odds ratio = 1.12 [95% CI: 1.01–1.24]; *P* = 0.026; **Figure 4**). Similar results were obtained using simple median, penalised weighted median, and weighted mode methods, with odds ratios of 1.19 (95% CI: 1.06–1.33), 1.14 (95% CI: 1.02–1.27), and 1.16 (95% CI: 1.02–1.32), respectively (**Figure 4**, **Supplementary Figure 5**). However, MR-Egger method yielded different result (**Figure 4**, **Supplementary Figure 5**). Heterogeneity was observed with Cochran’s Q test (Q: 54.2, *P* < 0.001), while MR-Egger intercept test revealed a nonsignificant intercept (*P* = 0.357). No outliers were identified in leave-one-out plot (**Supplementary Figure 11**). However, MR-PRESSO identified 1 outlier, yet its exclusion did not substantially impact the result (odds ratio = 1.17 [95% CI: 1.07–1.29]; *P* = 0.001). The results of the aforementioned MR analyses were summarized in **Supplementary Table 5**.

## 4 Discussion

Our findings provide several noteworthy findings: (1) NAFLD was independently associated with an increased risk of incident AVC in the observational analyses; (2) there was no consistent association between genetically predicted NAFLD and CAVS when considering all NAFLD susceptibility genes. However, after excluding genes associated with impaired VLDL secretion, the associations became significant between genetically predicted NAFLD and CAVS for all NAFLD-related traits, including cALT, imaging‐based and biopsy-confirmed NAFLD, as supported by multiple MR methods.

Previous studies have demonstrated a cross-sectional association between NAFLD and aortic valve sclerosis (13, 14). The observed association may be confounded by a series of coexisting cardiovascular risk factors, such as poor glycemic control, dyslipidemia and endocrine disorder (10, 34–36). In the present *post hoc* analysis of the MESA study, we confirmed the prospective association between NAFLD and an increased risk of incident AVC even after accounting for these potential confounding factors. These indicate that additional mechanisms beyond traditional cardiovascular and metabolic risk factors may contribute to this association. NAFLD exacerbated insulin resistance (37), caused atherogenic dyslipidemia, inflammation, and increased collagen synthesis, all of which have been implicated in the pathophysiology of valvular calcification (5). In addition, NAFLD also promoted ferroptosis (38), which was shown to be one of the main mechanisms of AVC (39). This further supports the possibility that the toxic systemic effects of NAFLD may be responsible for the observed association between NAFLD and the risk of AVC incidence.

In addition, some selected IVs (APOE, BCL7B, MTTP, TM6SF2, and PNPLA3) were reported to be related to impaired VLDL secretion, which directly affects blood lipid levels (40). After excluding these IVs, our two-sample MR analyses provide a novel perspective that NAFLD may causally impact the development of CAVS. The reasons for excluding genes related to impaired VLDL secretion were as follows. First, hyperlipidemia was recognized as a risk factor for CVD, and the above genetic variants also directly affected serum lipids, suggesting the possible influence of horizontal pleiotropy (40, 41). According to our results, when considering these IVs, the MR-Egger intercept test showed a statistically significant intercept. After exclusion of these IVs, the intercept became non-significant, indicating that we eliminated the influence of horizontal pleiotropy. In addition to considering the horizontal pleiotropy, we also excluded these IVs based on the biological reasons (42, 43). The stable isotope study has shown that increased flux of free fatty acids and higher rates of lipid synthesis are the primary causes of NAFLD (42). Furthermore, compared to individuals without NAFLD, participants with NAFLD typically exhibited an increase in VLDL secretion rather than a decrease (43).

This study has several strengths and limitations. First, to the best of our knowledge, it is the first to utilize two-sample MR analysis to examine the genetic association between NAFLD and CAVS. Second, we employed 3 gene‐exposure data of 3 different NAFLD‐related traits in combination with the use of different MR methods, which contributed to the robustness and validity of our findings. Notably, there are minor discrepancy in the results of the 3 different NAFLD-related traits. It was possibly because we identified SNPs associated with cALT based on GWAS. Thus, NAFLD susceptibility genes unrelated to cALT were not included in the MR analyses, which could affect our results. Additionally, the different sensitivities of the 3 different NAFLD-related traits to various histological stages of NAFLD may also contribute to these differences. Third, we used the latest and largest-scale available NAFLD GWAS results for the present analyses. Of note, the methodology of the foundational GWAS primarily identified NAFLD genes associated with cALT. It was likely that some NAFLD susceptibility genes, especially those unrelated to serum ALT levels, were omitted in our MR analysis. However, these NAFLD susceptibility genes were consistent with the imaging and biopsy evidence of NAFLD, suggesting that these selected genes are reliable NAFLD markers (28). Another limitation was that the original GWAS did not differentiate between the histological stages of NAFLD, a crucial distinction considering fibrosis’s specific association with cardiovascular mortality (44). Furthermore, while we excluded genes influencing NAFLD via impaired VLDL secretion, it did not guarantee the eradication of all potential horizontal pleiotropy, especially given that many of the considered SNPs exhibited expression beyond the liver.

In conclusion, our study confirmed that NAFLD was independently associated with an increased risk of incident AVC. The two-sample MR analyses further showed that genetically predicted NAFLD was also associated with CAVS incidence after excluding genetic variants related to impaired VLDL secretion. These findings underscore the relevance of the NAFLD in the development of CAVD and carry important implications for its prevention and treatment strategies.

## Data availability statement

The original contributions presented in the study are included in the article/**Supplementary Material**. Further inquiries can be directed to the corresponding authors.

## Author contributions

QH: Conceptualization, Formal analysis, Writing – original draft. YZ: Data curation, Formal analysis, Methodology, Writing – original draft. YL: Methodology, Writing – original draft. JG: Investigation, Writing – original draft. SL: Investigation, Writing – original draft. PY: Resources, Writing – original draft. JG: Supervision, Writing – review & editing. ZL: Conceptualization, Project administration, Writing – review & editing.
