# Supplementary material for: Observational and genetic association of non-alcoholic fatty liver disease and calcific aortic valve disease
Source: Front Endocrinol (Lausanne). 2024 Jul 9;15:1421642. doi: 10.3389/fendo.2024.1421642 (PMC11263017; doi:10.3389/fendo.2024.1421642)

**Supplementary Fig. 1.** Subgroup analysis of the associations between NAFLD and the risk of AVC incident. AVC, aortic valve calcification; BMI, body mass index; CI, confidence interval; HR, hazard ratio; NAFLD, non-alcoholic fatty liver disease.


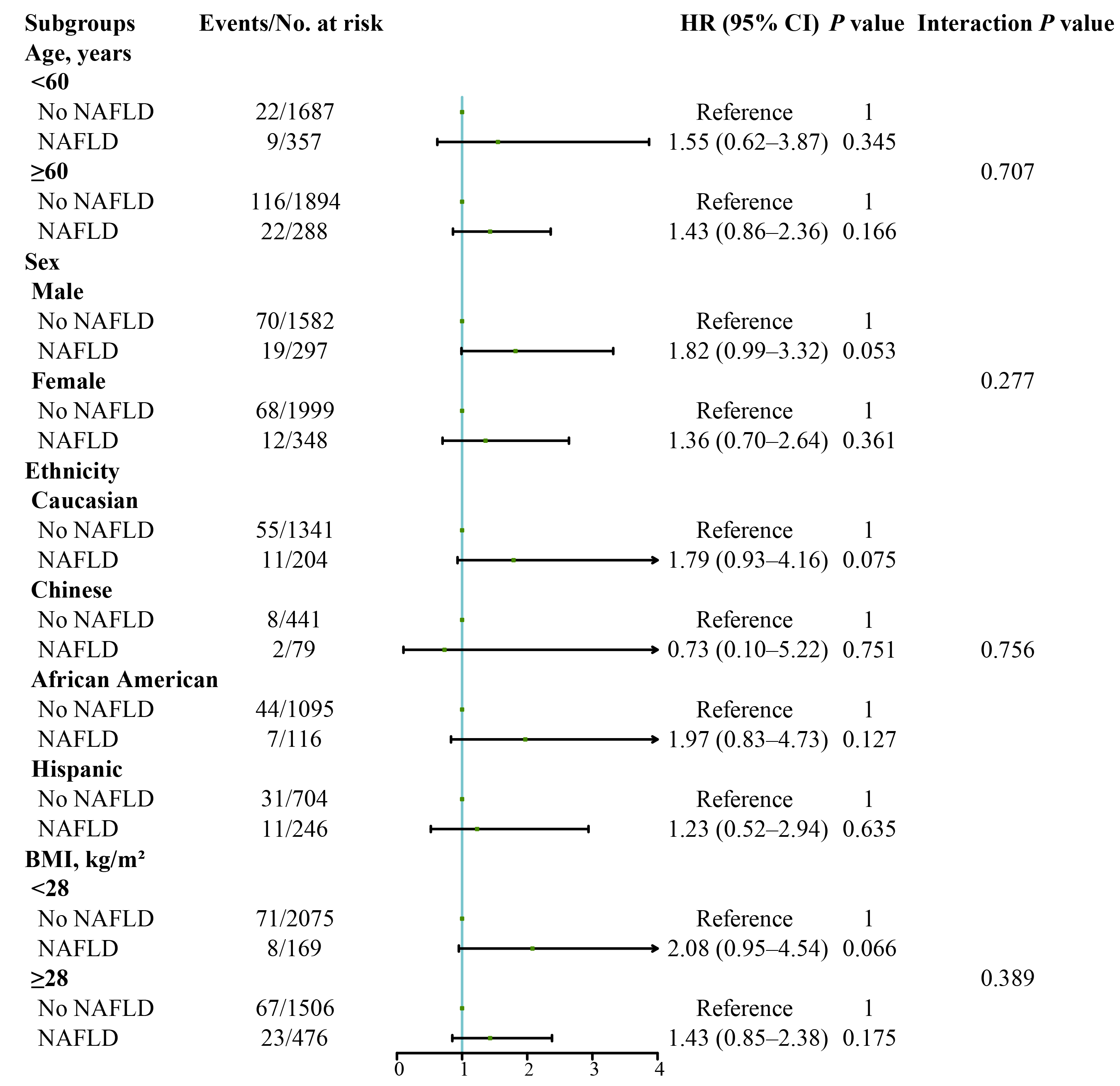


**Supplementary Fig. 2.** Scatter plots from genetically predicted imaging-confirmed NAFLD on CAVS. CAVS, calcific aortic valve stenosis; MR, Mendelian randomization; NAFLD, non-alcoholic fatty liver disease; SNP, single nucleotide polymorphism.


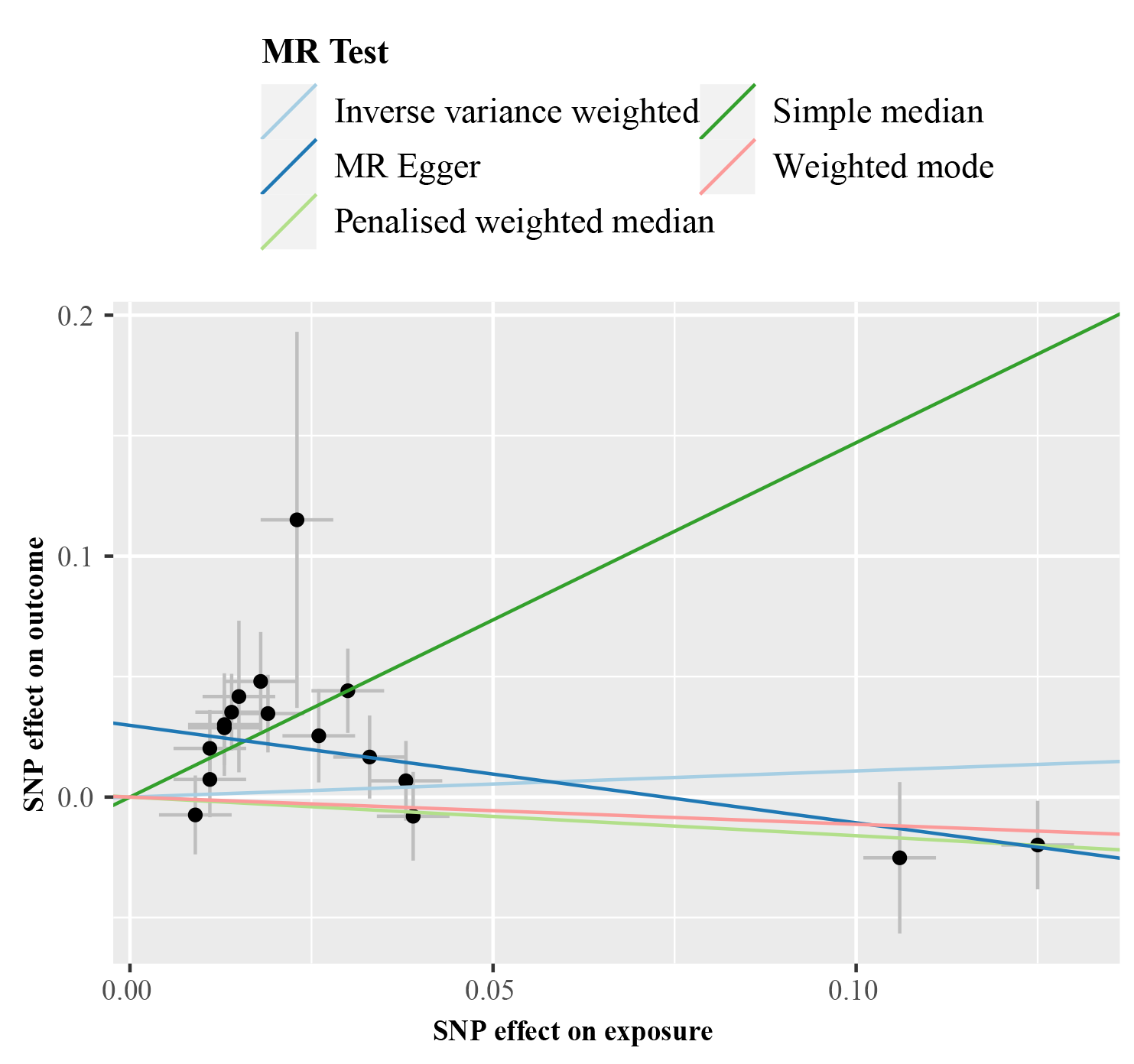


**Supplementary Fig. 3.** Scatter plots from genetically predicted biopsy-confirmed NAFLD on CAVS. CAVS, calcific aortic valve stenosis; MR, Mendelian randomization; NAFLD, non-alcoholic fatty liver disease; SNP, single nucleotide polymorphism.


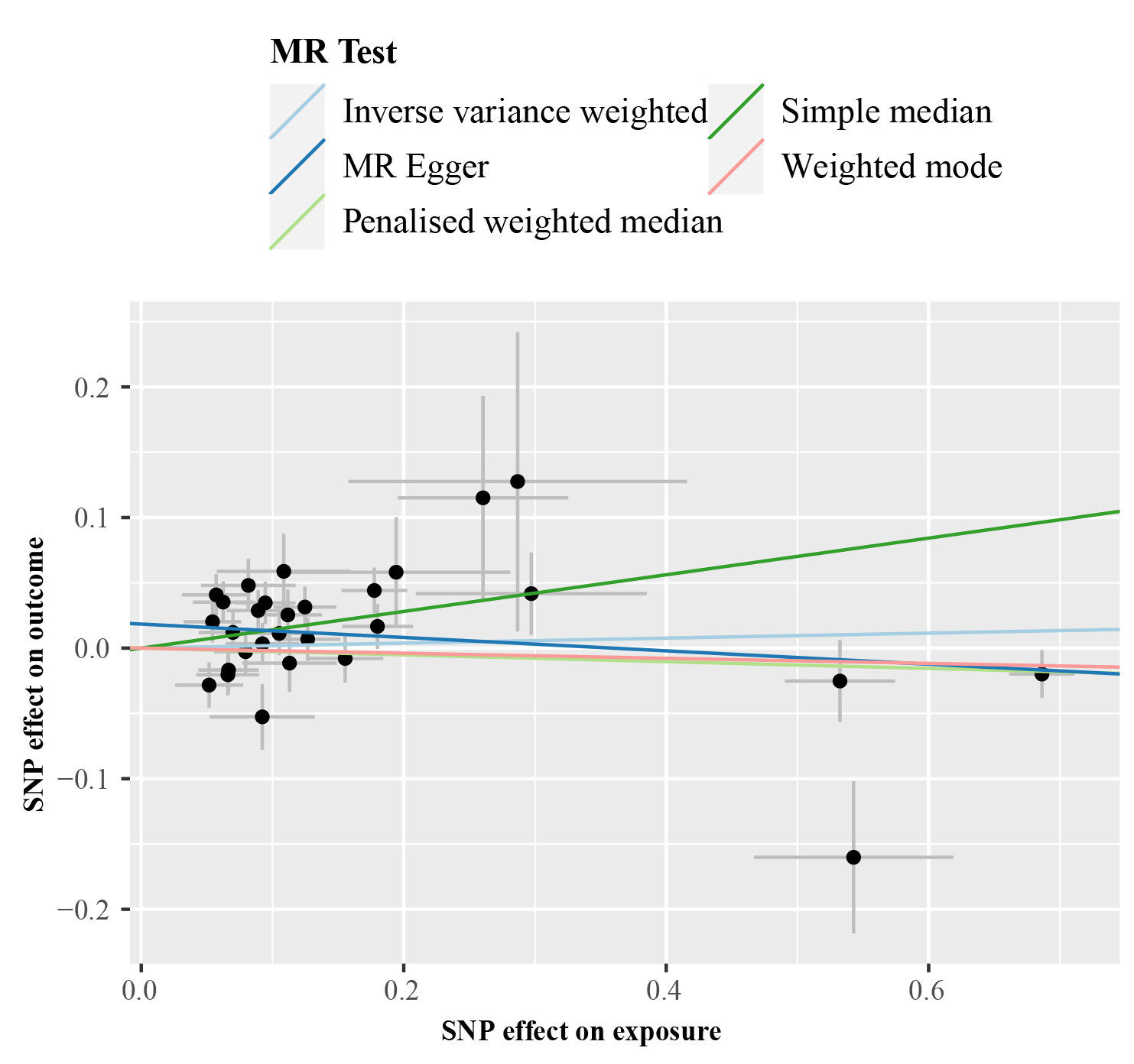


**Supplementary Fig. 4.** Scatter plots from genetically predicted imaging-confirmed NAFLD on CAVS after exclusion of genes associated with impaired VLDL secretion. CAVS, calcific aortic valve stenosis; MR, Mendelian randomization; NAFLD, non-alcoholic fatty liver disease; SNP, single nucleotide polymorphism; VLDL, very low-density lipoprotein.


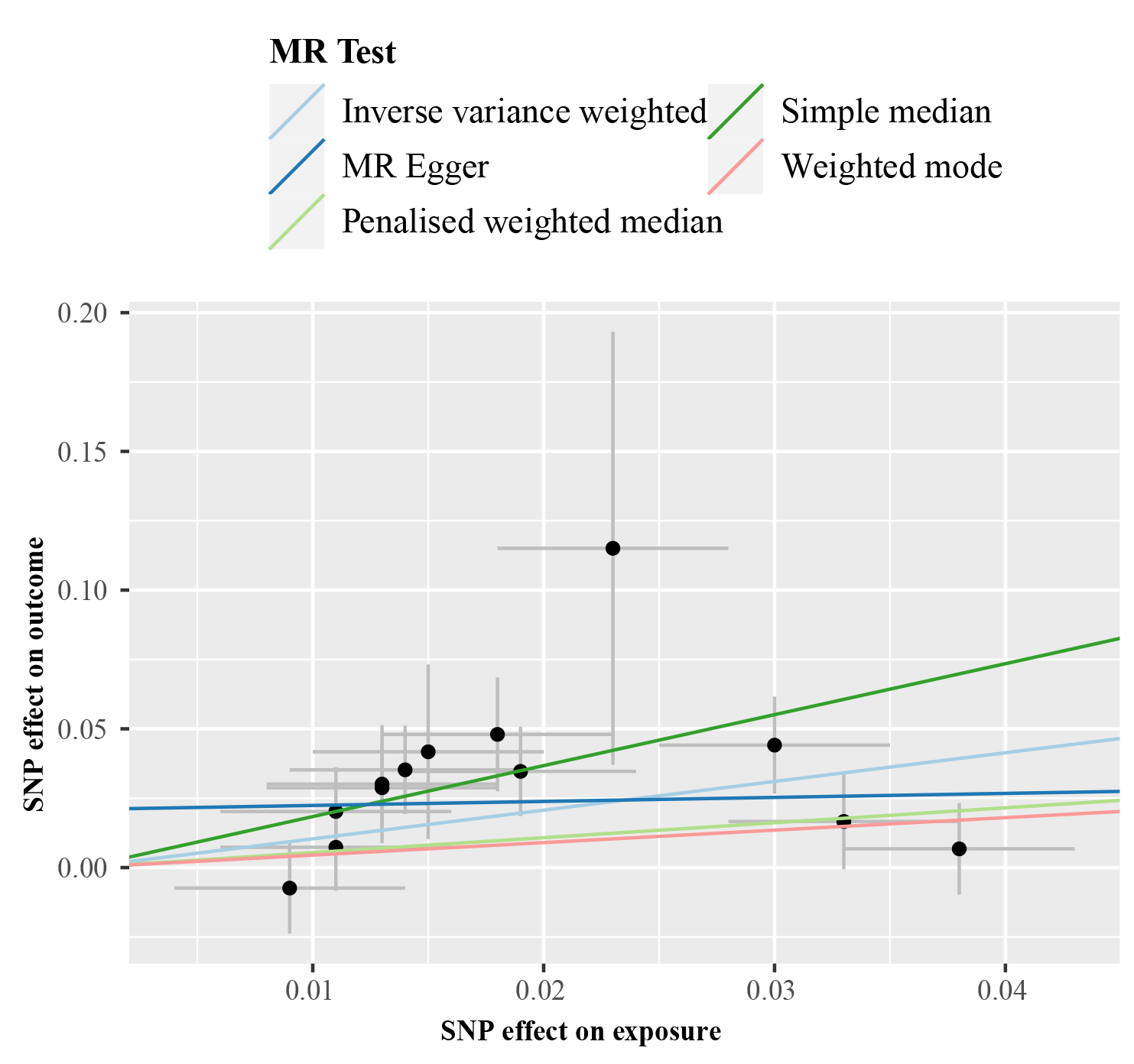


**Supplementary Fig. 5.** Scatter plots from genetically predicted biopsy-confirmed NAFLD on CAVS after exclusion of genes associated with impaired VLDL secretion. CAVS, calcific aortic valve stenosis; MR, Mendelian randomization; NAFLD, non-alcoholic fatty liver disease; SNP, single nucleotide polymorphism; VLDL, very low-density lipoprotein.


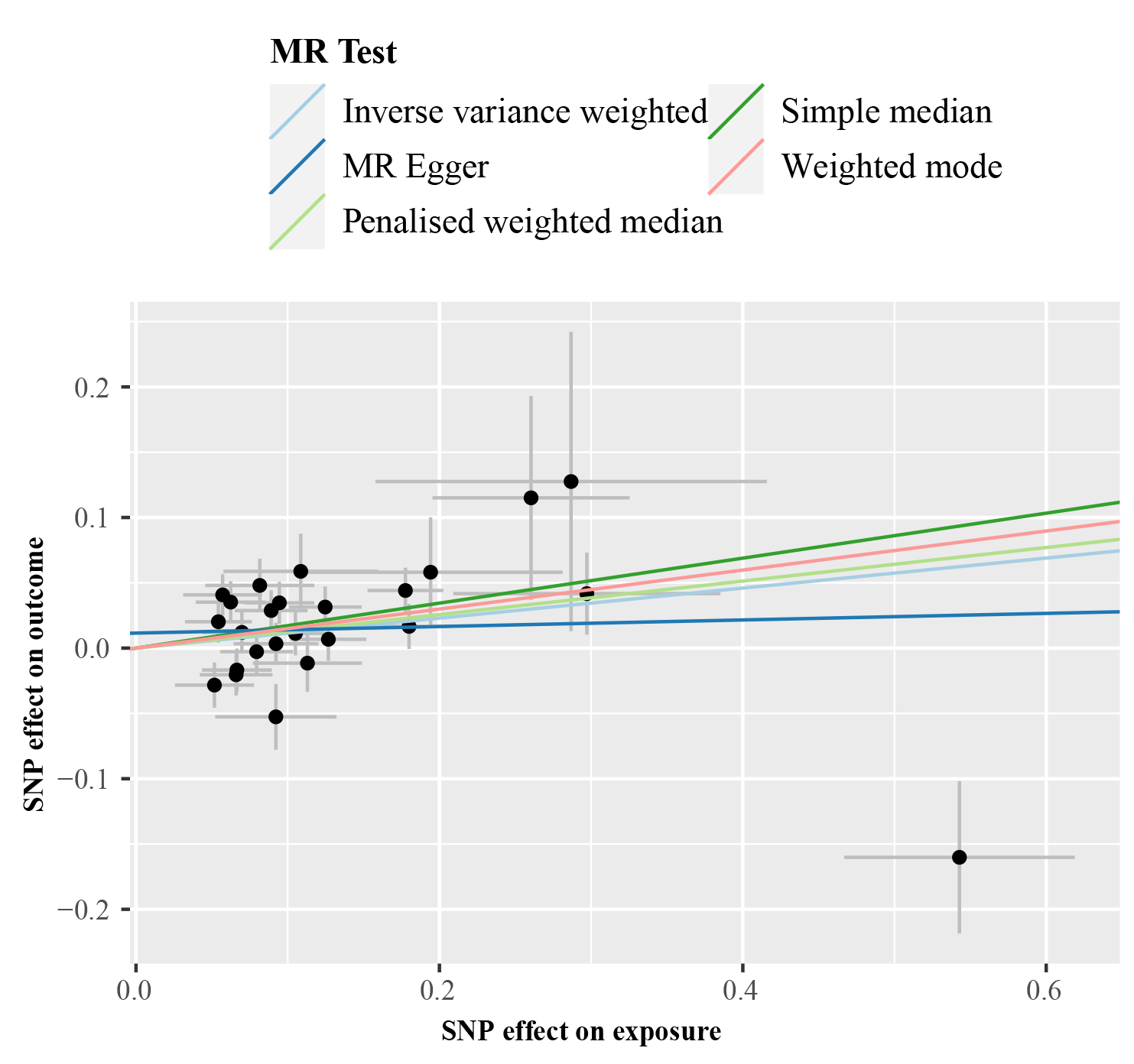


**Supplementary Fig. 6.** Leave-one-out plot from genetically predicted cALT on CAVS. cALT, chronic elevation of alanine transaminase; CAVS, calcific aortic valve stenosis; MR, Mendelian randomization.


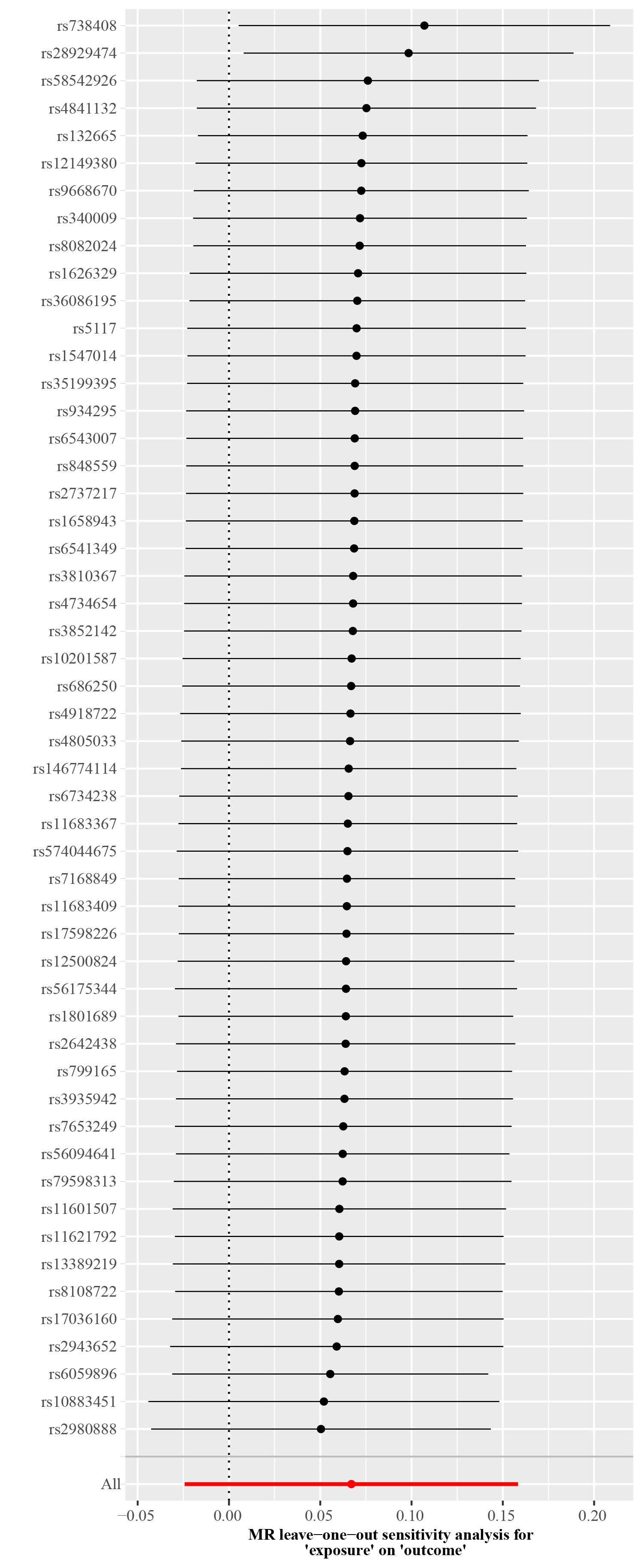


**Supplementary Fig. 7.** Leave-one-out plot from genetically predicted imaging-confirmed NAFLD on CAVS. CAVS, calcific aortic valve stenosis; NAFLD, non-alcoholic fatty liver disease; MR, Mendelian randomization.


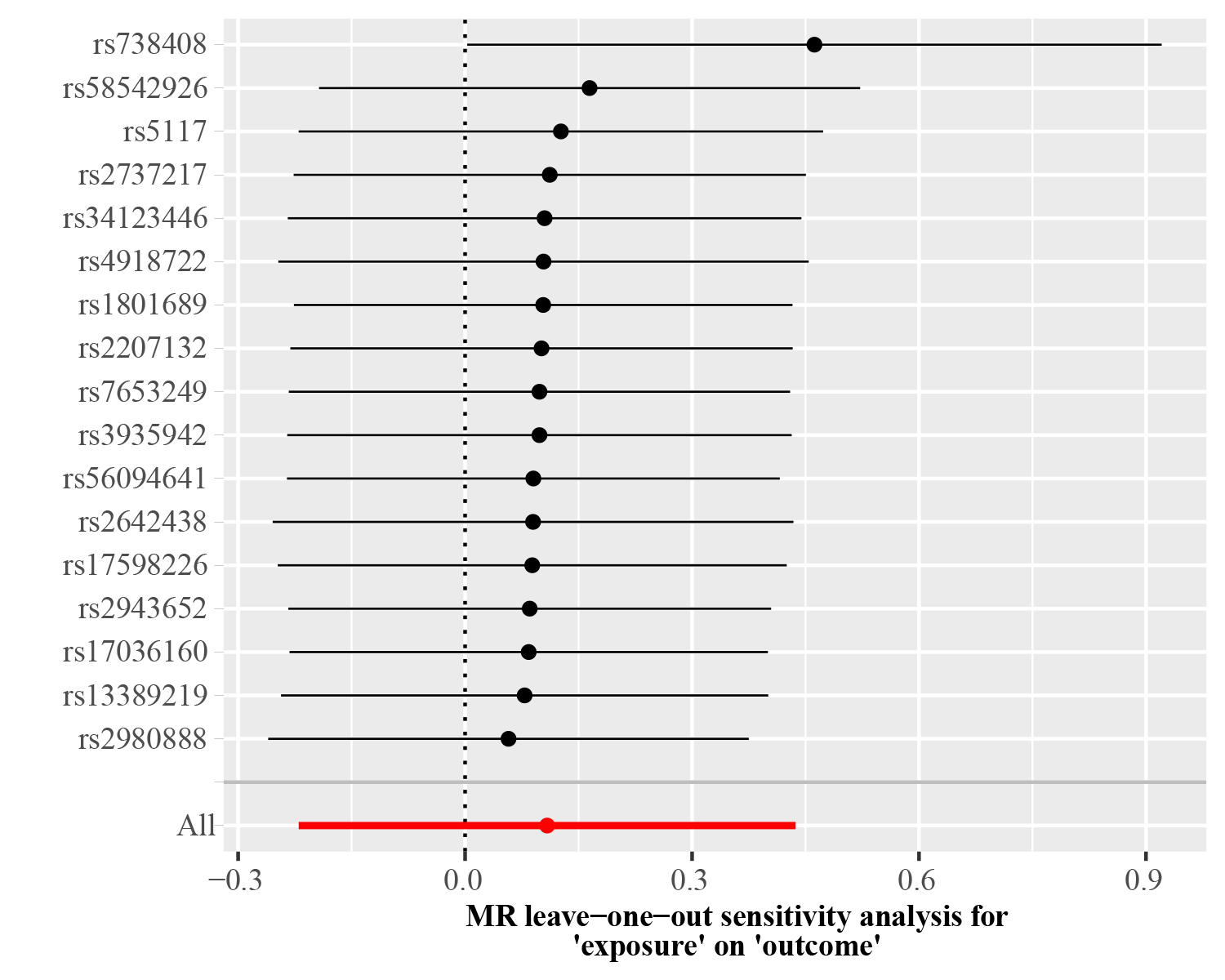


**Supplementary Fig. 8.** Leave-one-out plot from genetically predicted biopsy-confirmed NAFLD on CAVS. CAVS, calcific aortic valve stenosis; NAFLD, and non-alcoholic fatty liver disease; MR, Mendelian randomization.


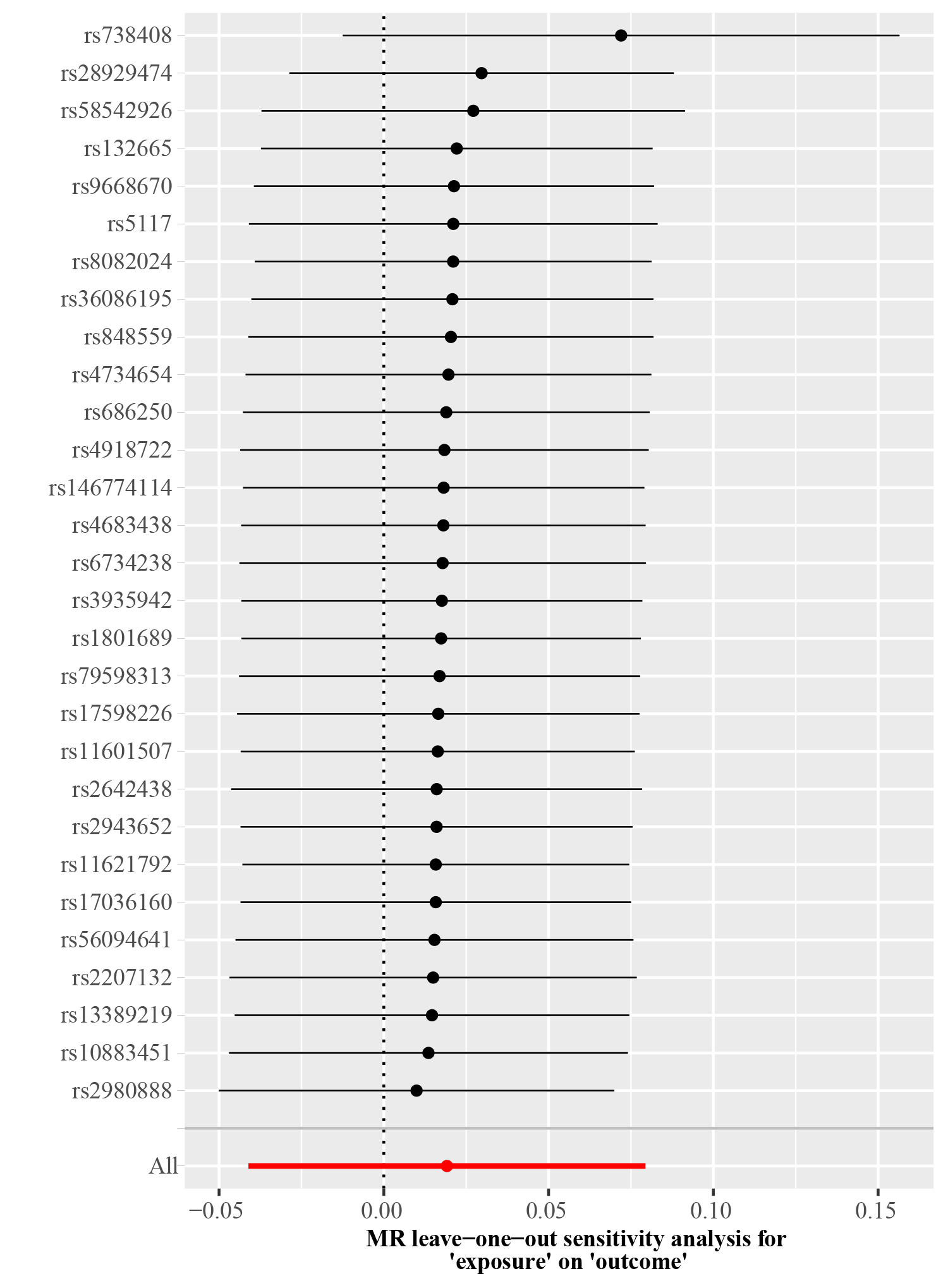


**Supplementary Fig. 9.** Leave-one-out plot from genetically predicted cALT on CAVS after exclusion of genes associated With impaired VLDL secretion. cALT, chronic elevation of alanine transaminase; CAVS, calcific aortic valve stenosis; MR, Mendelian randomization; VLDL, very low-density lipoprotein.


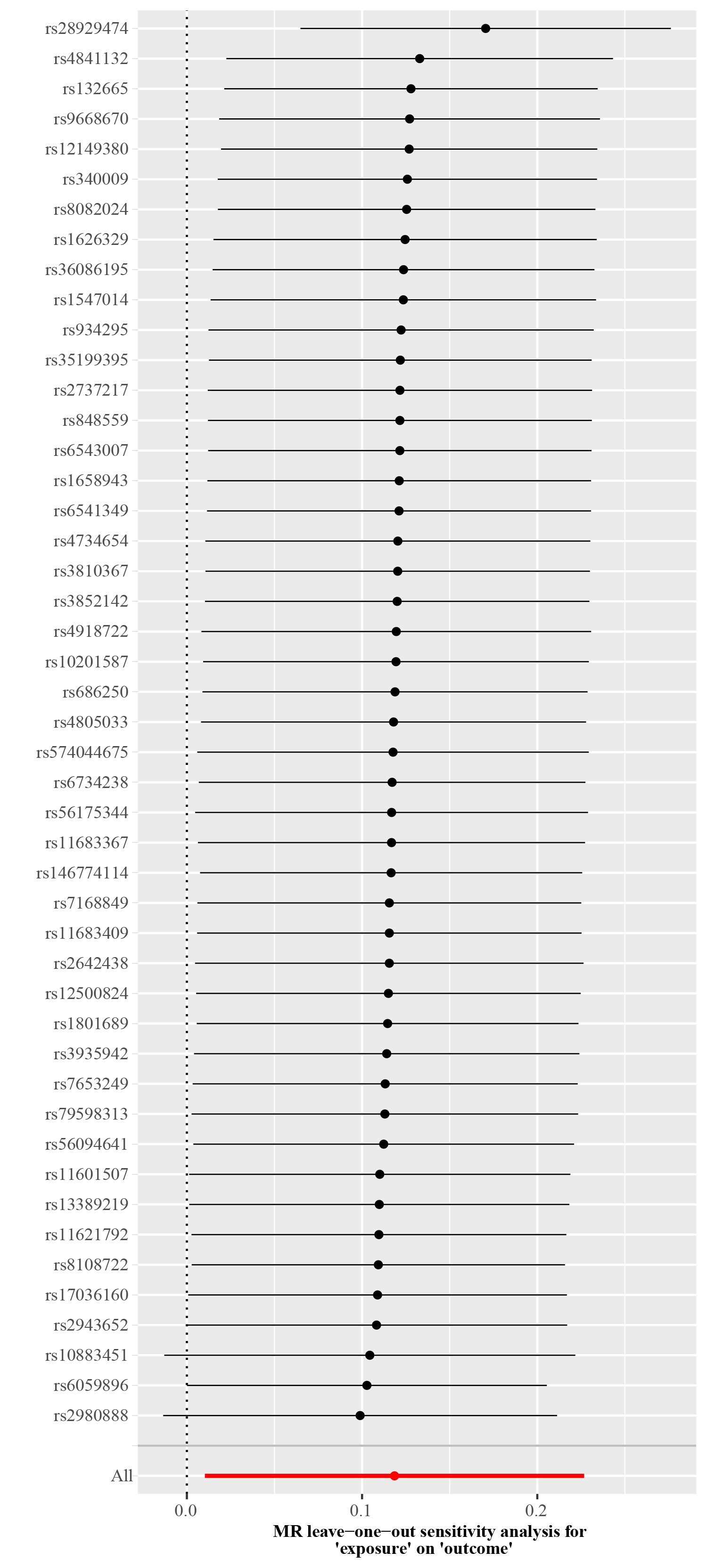


**Supplementary Fig. 10.** Leave-one-out plot from genetically predicted imaging-confirmed NAFLD on CAVS after exclusion of genes associated with impaired VLDL secretion. CAVS, calcific aortic valve stenosis; NAFLD, non-alcoholic fatty liver disease; MR, Mendelian randomization; VLDL, very low-density lipoprotein.


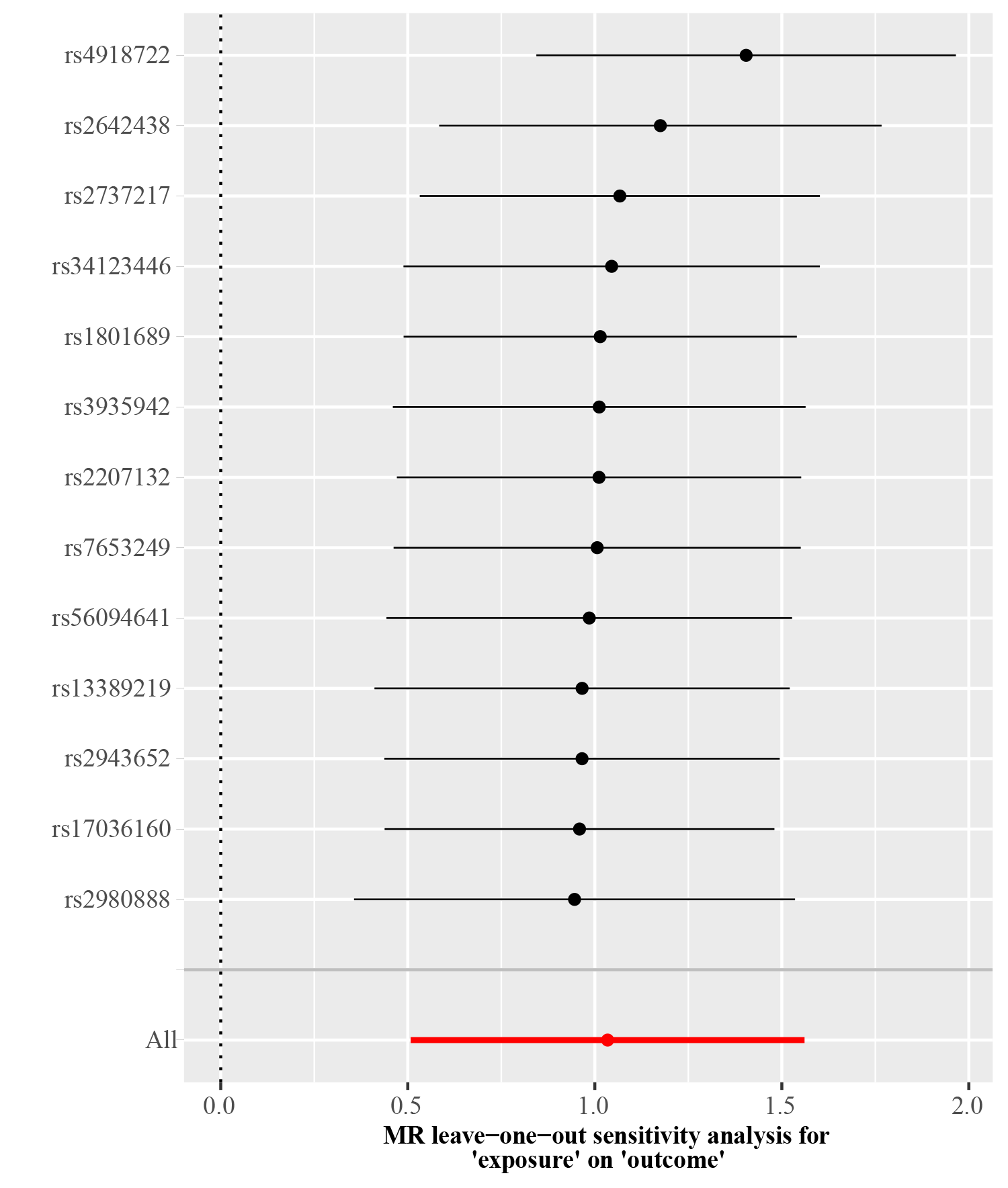


**Supplementary Fig. 11.** Leave-one-out plot from genetically predicted biopsy-confirmed NAFLD on CAVS after exclusion of genes associated with impaired VLDL secretion. CAVS, calcific aortic valve stenosis; NAFLD, non-alcoholic fatty liver disease; MR, Mendelian randomization; VLDL, very low-density lipoprotein.


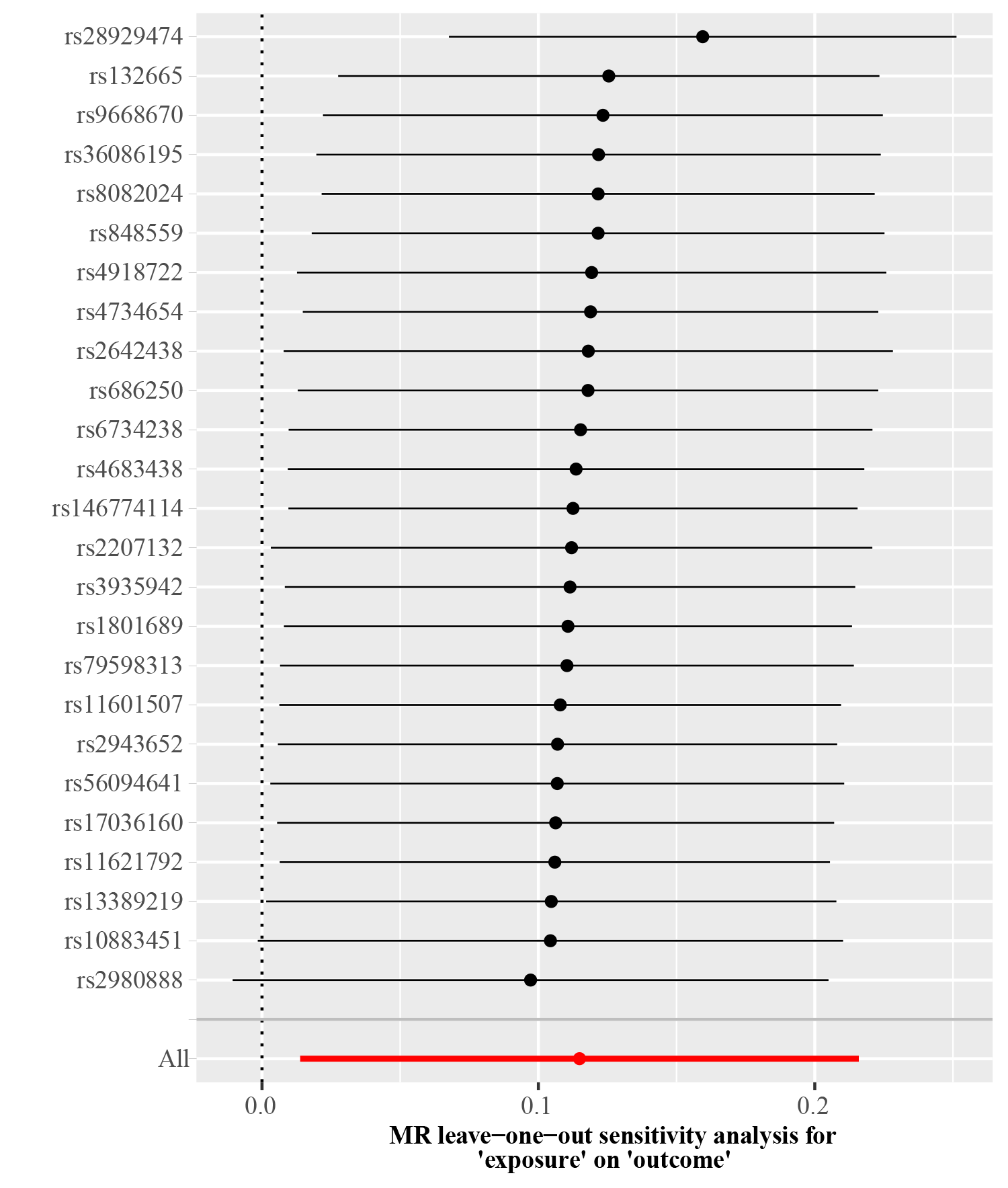

Supplement: Supplementary file 1 [file DataSheet_1.docx]
